# Dr. William Garner Sutherland (1873-1954): The Founder of Cranial Osteopathy

**DOI:** 10.7759/cureus.78705

**Published:** 2025-02-07

**Authors:** Brittany Childs, Cheryl Hammes

**Affiliations:** 1 Osteopathic Medicine, Ohio University Heritage College of Osteopathic Medicine, Warrensville Heights, USA; 2 Osteopathic Medicine, Family Medicine, Ohio University Heritage College of Osteopathic Medicine, Warrensville Heights, USA

**Keywords:** cranial osteopathic manipulative medicine, cranial osteopathy, cranio-sacral osteopathy, doctor of osteopathy, historical vignettes

## Abstract

Dr. William Garner Sutherland, a key figure in osteopathic medicine, significantly advanced the field through his development of cranial osteopathy, which has evolved into what is now termed osteopathic cranial manipulative medicine (OCMM). Born in rural Wisconsin in 1873, Sutherland was deeply influenced by Dr. Andrew Taylor Still, MD, DO, the founder of osteopathy, during his studies. Inspired by Still's emphasis on the body's natural healing abilities, Sutherland proposed that the cranial bones are capable of subtle movements essential for health, a theory he detailed in his seminal work, *The Cranial Bowl*. His concept of primary respiratory movement (PRM) and the role of the sphenobasilar synchondrosis revolutionized osteopathic medicine, linking cranial bone mobility to overall well-being. Despite initial skepticism, Sutherland's ideas inspired future practitioners such as Harold Magoun, who further developed and disseminated OCMM. This review aims to synthesize historical and academic perspectives on Sutherland’s contributions, analyzing his development of OCMM, its theoretical foundations, and its lasting impact on osteopathic education and practice. Given that Sutherland did not produce a personal biography during his lifetime, this work synthesizes information from his posthumous volume, *With Thinking Fingers* (1962), authored by his spouse, Adah Strand Sutherland, alongside a comprehensive literature search of peer-reviewed journals, textbooks, and published works. Key objectives include documenting Sutherland's formative years and academic influences, examining his role in evolving Still’s principles, and evaluating his research in OCMM. His legacy persists through institutions such as the Sutherland Cranial Teaching Foundation (SCTF), the Osteopathic Cranial Academy, and the Osteopathic Center for Children, all of which continue to promote his methodologies and ensure the enduring influence of his work in osteopathic medicine.

## Introduction and background

Early life

**Figure 1:**
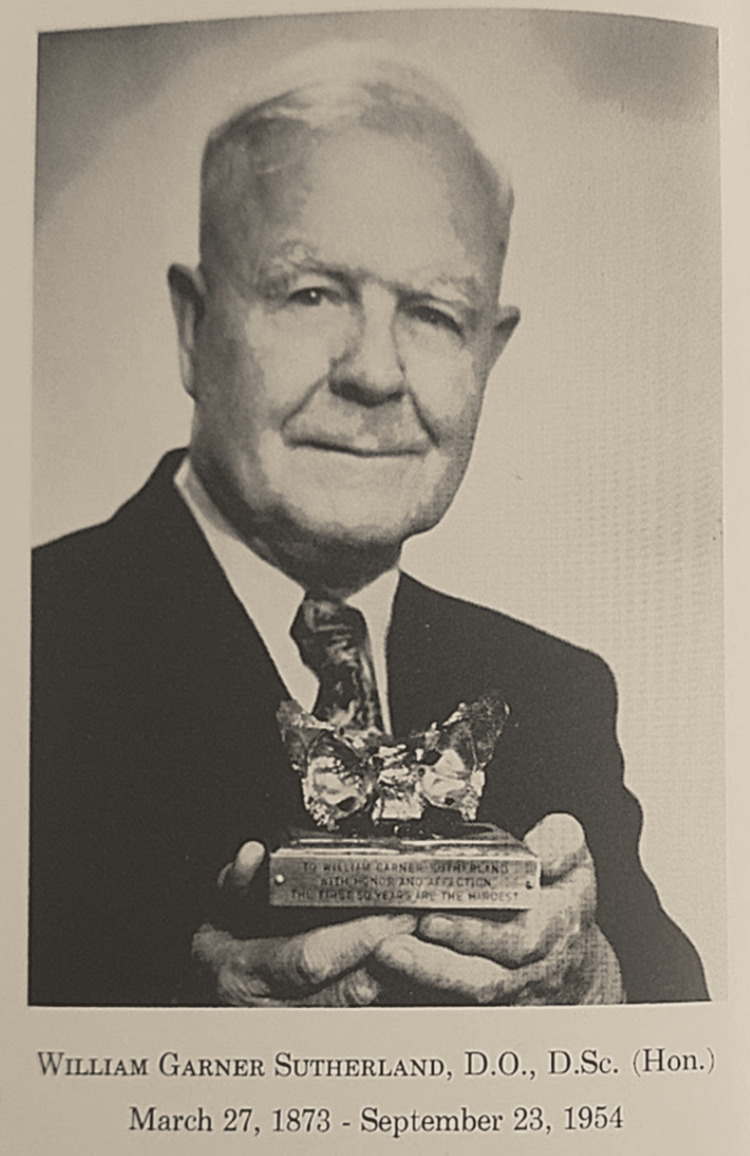
Portrait of Dr. Sutherland from the book With Thinking Fingers, written by his wife, Adah Sutherland. Source: HathiTrust Digital Library William Garner Sutherland, D.O., D.Sc. (Hon.) March 27, 1873 - September 23, 1954. pg. vi (https://babel.hathitrust.org/cgi/pt?id=rul.33008005924372&seq=10)

Dr. William Garner Sutherland (1873-1954) was born on March 27, 1873, in Portage County, Wisconsin, a region characterized by its rural landscape and agricultural economy. The environments of his upbringing in Wisconsin, Minnesota, and South Dakota, rich in natural beauty and simplicity, likely influenced his later appreciation for the body's natural functions and its capacity for self-regulation. Our understanding of Sutherland’s early life is primarily based on accounts compiled by his wife, Adah Strand Sutherland, who integrated portions of his personal notes into her work, *With Thinking Fingers.* The second child of four, Sutherland (Figure 1) was born to Robert and Dorinda Sutherland. Though his father labored as both a blacksmith and lumberjack, his earnings were often insufficient to support the family as he wished. By the age of 14, William had left school to contribute to the family finances, securing a job at the local newspaper, *The Blunt Advertiser*. When the publisher moved to another newspaper, William followed him, leading to several job changes in print; until in 1891, he landed at The Mapleton Enterprise in Mapleton, Minnesota, as a foreman. Throughout this time, Mrs. Sutherland writes that he yearned for higher education. Eventually, he would go on to college in Iowa, although he left without a diploma returning to scholastic printing in Minnesota. Around 1895, it was there that he first learned of Dr. Still and his groundbreaking "cure," osteopathy [1].

Advances in early 19th-century medicine ushered in a significant shift in disease conceptualization and medical treatment. Pathological anatomy, a pivotal development, redefined disease understanding, and by 1858, Rudolf Virchow’s focus on cellular pathology further deepened this knowledge, establishing a more precise basis for diagnosing illness. [2]. Before this, symptoms such as fever, diarrhea, vomiting, and respiratory issues were often mistaken for the disease. Autopsy findings from this period also began to reveal pathological changes within organs as the true cause, paving the way for a more accurate understanding of illness. This background may have played a role in shaping Sutherland's interest in exploring alternative approaches to symptomatic dysfunction. Sutherland eventually pursued his education at the American School of Osteopathy (ASO) in Kirksville, Missouri, where he became a student of Dr. Andrew Taylor Still, the founder of osteopathy [3]. This institution was the first of its kind, focusing on the principles of osteopathy that Still had developed. Sutherland's time at ASO marked a significant turning point in his life, as he became deeply influenced by Still's teachings and philosophy, which emphasized the body's ability to heal itself when in proper musculoskeletal alignment. This foundational education under Still set the stage for Sutherland's future innovations in osteopathy, particularly around cranial manipulation.

The beginnings of Andrew Taylor Still and the birth of osteopathic medicine

**Figure 2:**
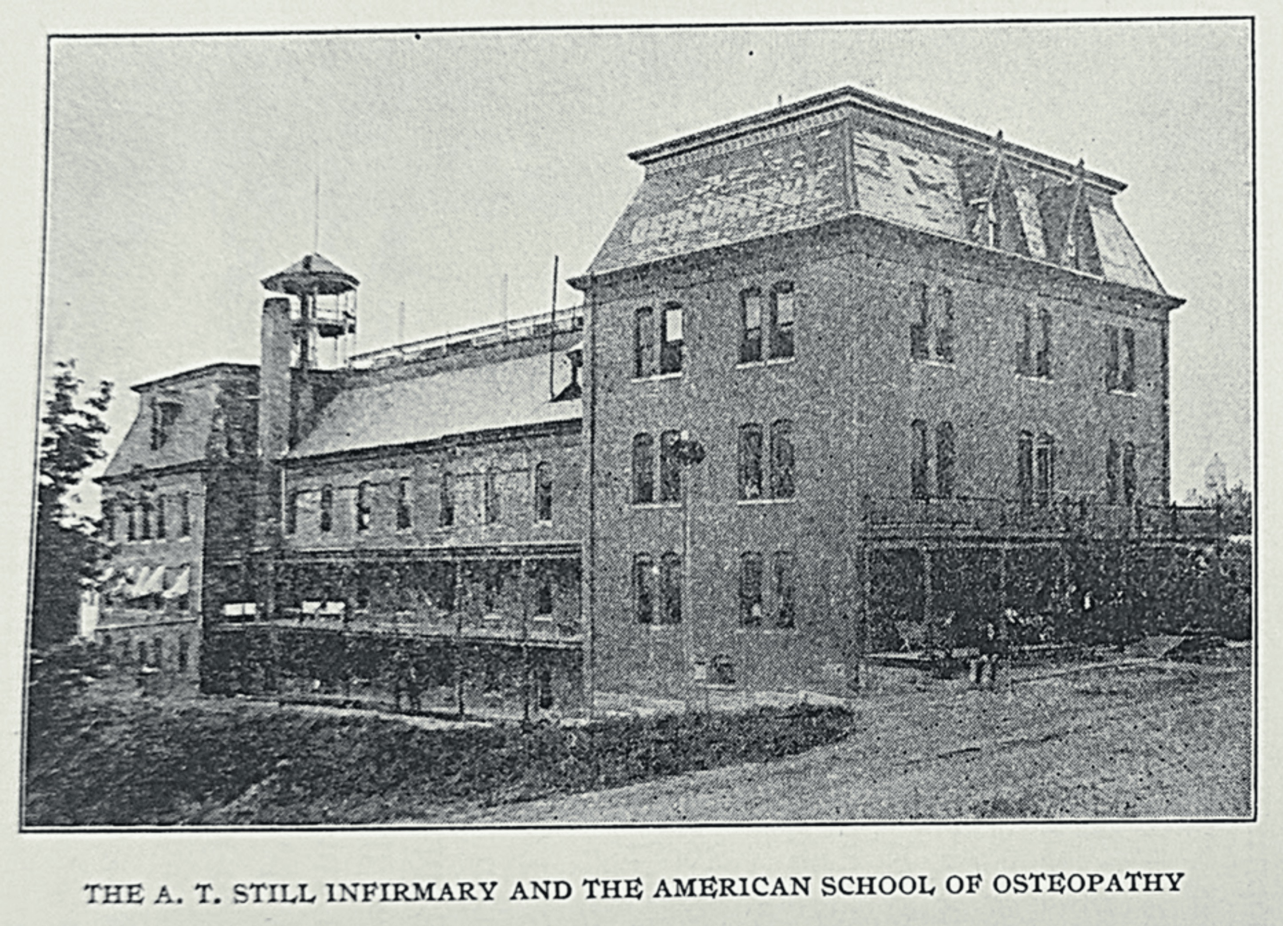
Photograph of the world's first osteopathic medical school. Source: Autobiography of Andrew T. Still The A. T. Still Infirmary and the American School of Osteopathy. pg. 143 (https://babel.hathitrust.org/cgi/pt?id=chi.24129195&seq=199)

Given the circumstances, Still's influence on Sutherland cannot be overstated, as it provided the foundation upon which Sutherland built his own theories and practices. Dr. Andrew Taylor Still (1828-1917) is widely regarded as the father of osteopathic medicine, a medical practice that emerged in the late 19th century as an alternative to the often harsh and invasive medical treatments of the time. Notably, his early understanding of osteopathy was shaped by a childhood experience in which he described relieving a headache and sick stomach by positioning his neck between a rope tied between two trees [4]. While trained as an allopathic physician, he became disillusioned with conventional medicine after seeing the deaths of two of his wives from pneumonia and childbirth and three of his children from spinal meningitis. This personal tragedy spurred him to develop an original approach to healthcare, one that emphasized the body's inherent ability to heal itself. Still's groundbreaking efforts resulted in the founding of the first osteopathic school (Figure 2) in Kirksville, Missouri, where he passed on his principles to a new generation of osteopathic physicians, known as Doctor of Osteopathic Medicine (DOs), including William Sutherland. In the formal constitution filed on October 30, 1894, records detail the name and style of the corporation with "an object to improve our present system of surgery, obstetrics, and treatment of diseases generally, and place the same on a more rational and scientific basis..." [4].

Still's approach to medicine was revolutionary for its time, focusing on the interrelationship between the body's structure and function. He believed that many diseases were caused by structural problems in the musculoskeletal system that impeded the body's natural healing processes [5]. By manipulating these structures, particularly the spine, Still believed that he could restore health and prevent illness. This philosophy resonated deeply with Sutherland, who was inspired to expand on Still's ideas and apply them to the cranial bones.

Early observations and theories

**Figure 3:**
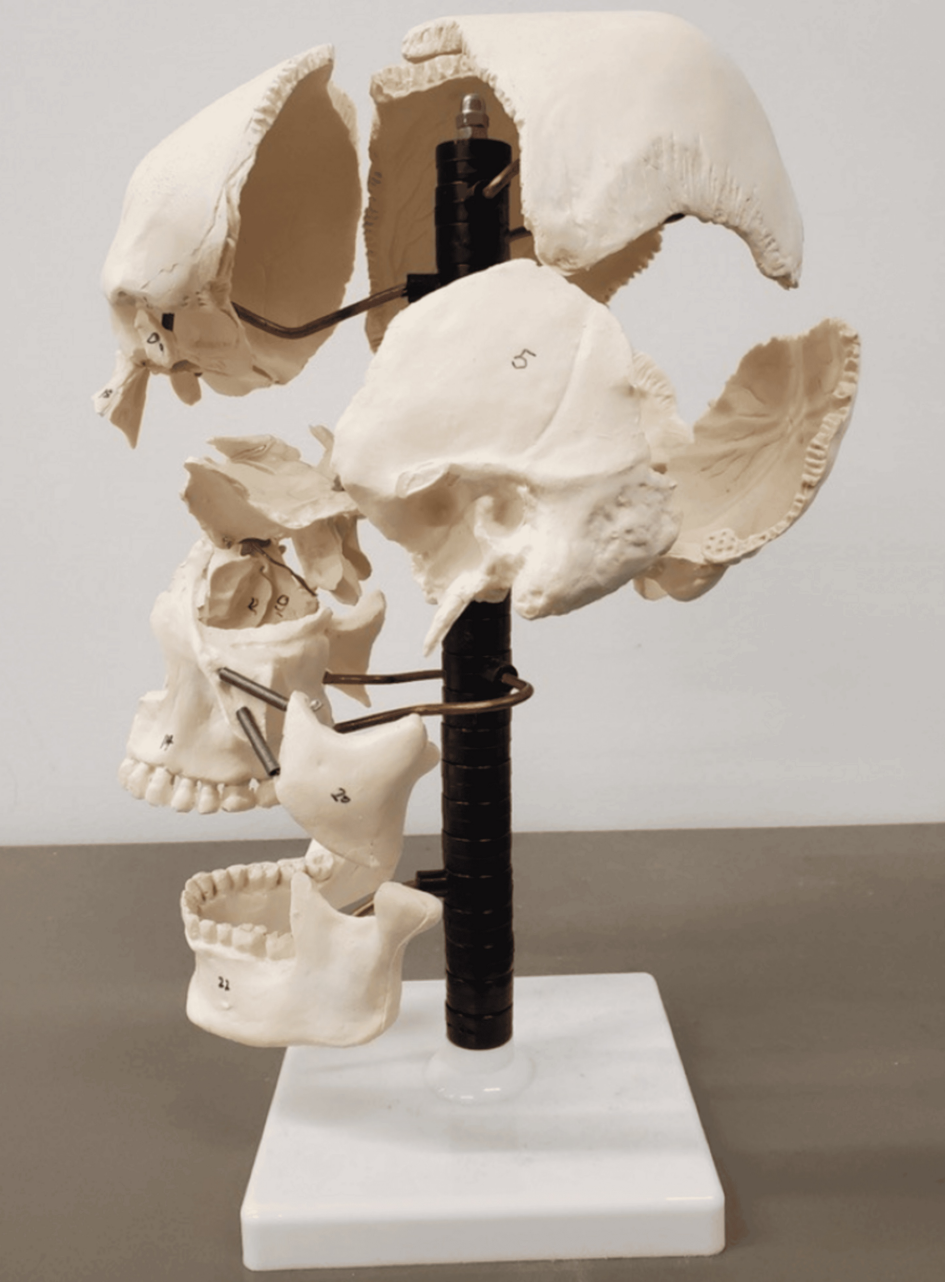
Surgeon and anatomist Edmé François Chauvot de Beauchêne invented in the 1810s the technique of taking apart the individual bones of the skull and reassembling them in an “exploded” fashion on a frame for better viewing. Source: Unpublished faculty photograph, Ohio University Heritage College of Osteopathic Medicine Universal CC-BY access

Around 1898, Dr. Sutherland's journey into cranial osteopathy began with a curious observation while he was still a student at the ASO (now the Kirksville College of Osteopathic Medicine). While examining a disarticulated skull (Figure 3), he noticed that the cranial bones were beveled, as though designed for a respiratory-like movement [6]. This observation sparked a series of experiments and self-reflections that led Sutherland to hypothesize that the cranial bones were, in fact, capable of movement and played a crucial role in the body's overall health. This idea was revolutionary at the time, as the prevailing medical consensus held that adult skull bones were fused and immovable. Fortunately, for modern medicine, he did not let his self-ridicule, disgust, and self-argument on this novel concept hold him back, as these thoughts would eventually lead to the development of cranial osteopathy.

One of the most famous stories about Sutherland's early explorations involves a leather helmet he fashioned to test his theories on cranial bone movement. By tightening and loosening the straps of the helmet, he applied varying pressures to his own skull, noting the physical and mental effects of these adjustments [7]. His experiments led him to develop the concept of cranial rhythmic impulse, a subtle, rhythmic movement within the cranial bones that he believed was essential to the body's health. Dr. Sutherland's theories were later compiled in his seminal work,* The Cranial Bowl *(1994), which laid the groundwork for the practice of cranial osteopathy, now called osteopathic cranial manipulative medicine (OCMM). His work was both groundbreaking and controversial, challenging the established norms of anatomy and physiology at the time. Notably, the book also reflects the personal side of his career, including his mother-in-law’s appreciation for his work.

There is a reason for each move of the osteopathic intelligent fingers; and each move of the fingers is governed by reason. - Mother Anice Gault Strand [6].

**Figure 4:**
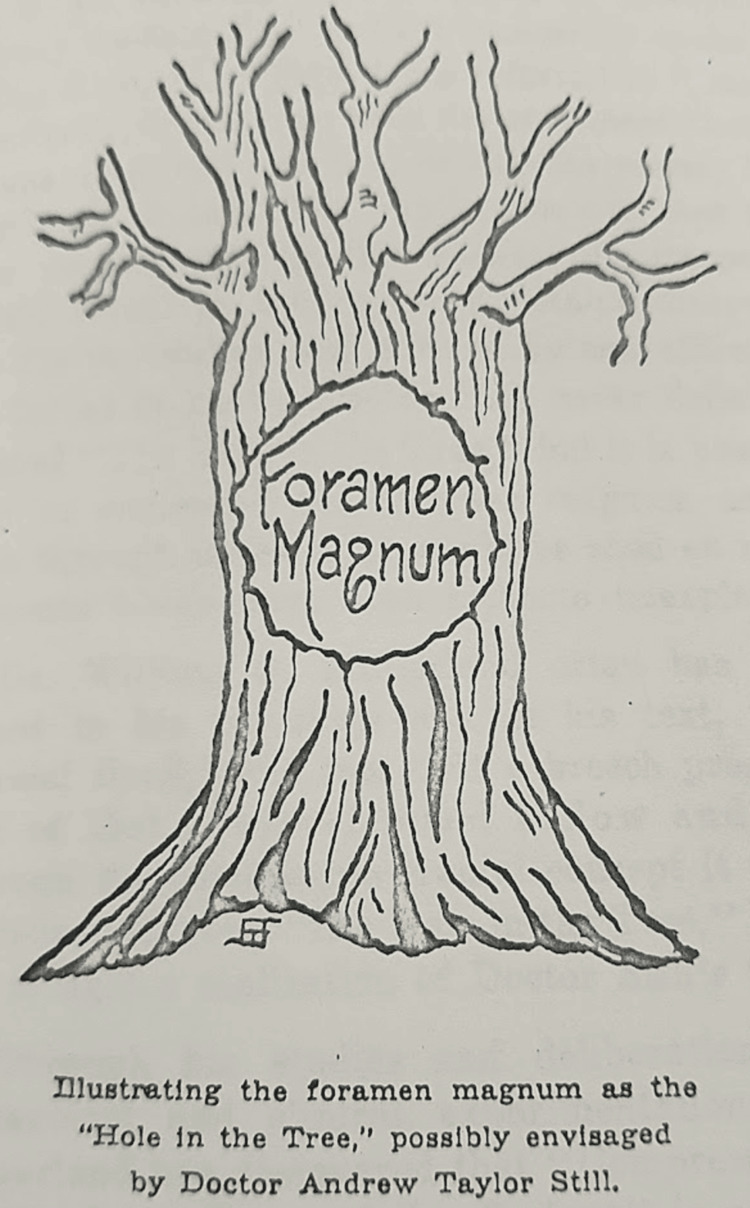
Foramen magnum "hole in the tree" analogy illustration from Sutherland's book, The Compression of the Condylar Parts of the Occiput. Source: Sutherland, W. G., Lippincott, H. A., & Lippincott, R. C. (1945). Compression of the Condylar Parts of the Occiput. Free Press. Public domain

An additional theory would be based on what Dr. Still often spoke of, "the hole in the tree," a metaphor that he used to represent a yet undiscovered part of osteopathy that would eventually reveal crucial insights. It is speculated that this "hole" referred to the foramen magnum (Figure 4), the large opening at the base of the skull where the brain meets the spinal cord. In *The Compression of the Condylar Parts of the Occiput*, Dr. Sutherland used a powerful analogy, comparing the challenging birth of certain animals to the process of cranial osteopathy, suggesting that they could be "delivered from The Hole in the Tree." This metaphor aligns with his broader understanding of cranial function, where he viewed the skull as a dynamic structure capable of influencing overall health. Sutherland also discussed the concept of "bent twig" skulls he encountered in his practice, such as the malleable skulls of newborns, which are influenced by the compressive forces of labor and delivery, potentially impacting cranial mobility and development. These deformations, he believed, were akin to the "twisting" of branches in human growth and development, leading to dysfunction later in life [8]. He is said to have recounted this proverbial phrase, "As the twig is bent, so the tree doth incline," underscoring his belief that early structural imbalances could profoundly shape the body’s functional form as it develops. Sutherland’s use of natural metaphors like these helped convey the deep connection he saw between the body’s form and its capacity for self-regulation and healing.

The booklet, prepared in collaboration with Drs. Howard and Rebecca Lippincott, was the culmination of Sutherland’s years of research. Created during a working vacation by Lake Superior, it became a critical reference in the field of osteopathy, particularly for advanced cranial courses. The illustration of the foramen magnum as the "hole in the tree" served as a visual representation of the profound anatomical significance that both Still and Sutherland attached to the cranial anatomy in osteopathy [8]. Today, with our understanding of the myodural bridge connecting the dura mater to the upper cervical spine, the anatomical insights proposed by Sutherland and Still find new relevance, underscoring their anticipation of concepts later confirmed by science.

## Review

Skull bone mobility and cranial breathing 

The concept of primary respiratory movement (PRM) is central to OCMM [6]. Initially, the joints of the cranium were misunderstood, as they had traditionally been described based on observations from cadavers rather than living subjects. Sutherland proposed that the movement of the cranial bones was linked to the circulation of cerebrospinal fluid (CSF) and that this movement was vital for the body's health.

This theory is based on five key principles mentioned in Table 1 [9,10].

**Table 1:** Five principles of primary respiratory movement (PRM). Sources: Refs. [9,10]

Five Principles of PRM	Description
Fluctuation of Cerebrospinal Fluid (CSF)	The rhythmic motion of CSF, essential for nutrient delivery and waste removal in the central nervous system.
Inherent Motility of the Central Nervous System and Spinal Cord	Subtle, intrinsic movement of the brain and spinal cord, independent of respiratory or cardiac motion.
Mobility of the Meningeal Membranes	The dura mater and other meningeal structures facilitate cranial motion and cerebrospinal fluid circulation.
Joint Mobility of the Cranial Bones	Contrary to previous beliefs, cranial sutures allow for small but important movements that influence overall health.
Involuntary (Passive) Movement of the Sacrum Between the Iliac Bones	The sacrum moves in response to cranial motion, reflecting the interconnected nature of the body's structural and functional systems.

The sphenobasilar synchondrosis (SBS), the joint between the base of the occiput and the body of the sphenoid bone, was identified by Sutherland as a key pivot point for cranial motion. He believed that the mobility of the SBS was crucial for the proper function of the central nervous system and, by extension, the entire body.

**Figure 5:**
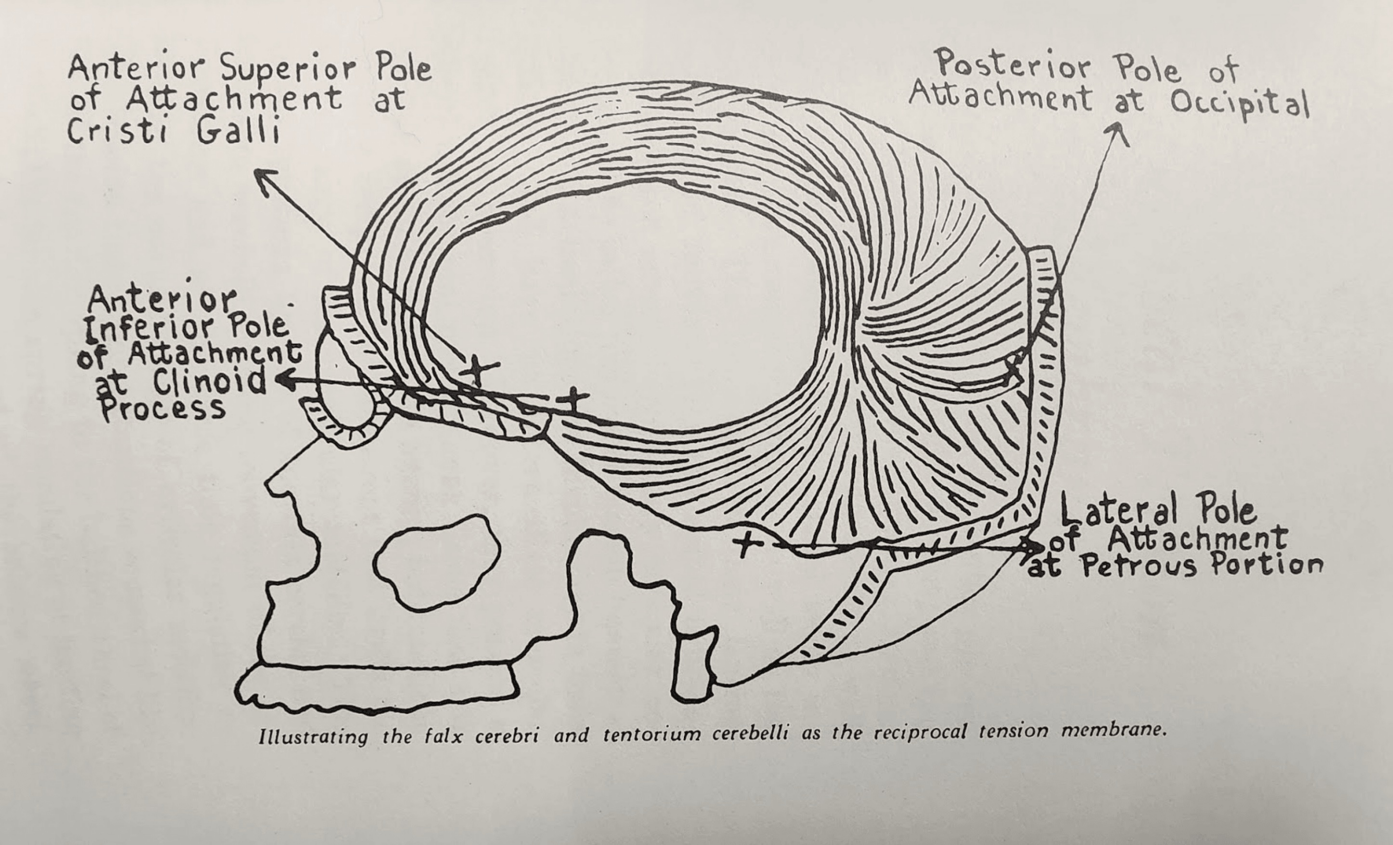
Image from the book "The Cranial Bowl" to illustrate the attachment points of the falx cerebri and tentorium cerebelli as components of the reciprocal tension membrane within the cranial cavity. Source: Ref. [6] Open-access publication

In his foundational work, Dr. Sutherland describes the intricate dynamics of cranial articulation through the concept of the reciprocal tension membrane (Figure 5). This system, composed of the falx cerebri and tentorium cerebelli, plays a crucial role in regulating intracranial mobility. By acting as a dynamic tension regulator, the reciprocal tension membrane facilitates proper articulation between the cranial bones, allowing for a rhythmic and balanced movement during respiration. As noted by Sutherland, the anterior superior pole of the crista galli and the posterior occipital attachment demonstrate finely tuned coordination. During inhalation, the anterior portion moves anteriorly and superiorly, while the posterior portion shifts posteriorly and inferiorly. Conversely, during exhalation, this motion reverses, ensuring that the cranial bones maintain their structural integrity while preserving mobility within a normal physiological range. The harmony of this mechanism is reminiscent of a finely balanced watch spring, carefully regulating motion to preserve the overall function of the cranial system.

Sutherland also explored the connection between cranial movement and the visceral system, suggesting that restrictions in cranial bone mobility could have far-reaching effects on the body's organs and overall health. This holistic view of the body, where the cranial bones, CSF, and visceral systems are interconnected, was a significant departure from the more mechanistic views of the body prevalent at the time. Sutherland's work laid the foundation for further exploration into how the cranial bones and the body's internal systems interact, influencing the development of various osteopathic techniques aimed at restoring balance and health through cranial manipulation.

The skull is fastened to the spine And often hides an aching brain; A DO has found the way To move the bones and ease the pain. - Mother Dorinda Smith Sutherland [6]

Inspirational impact

**Figure 6:**
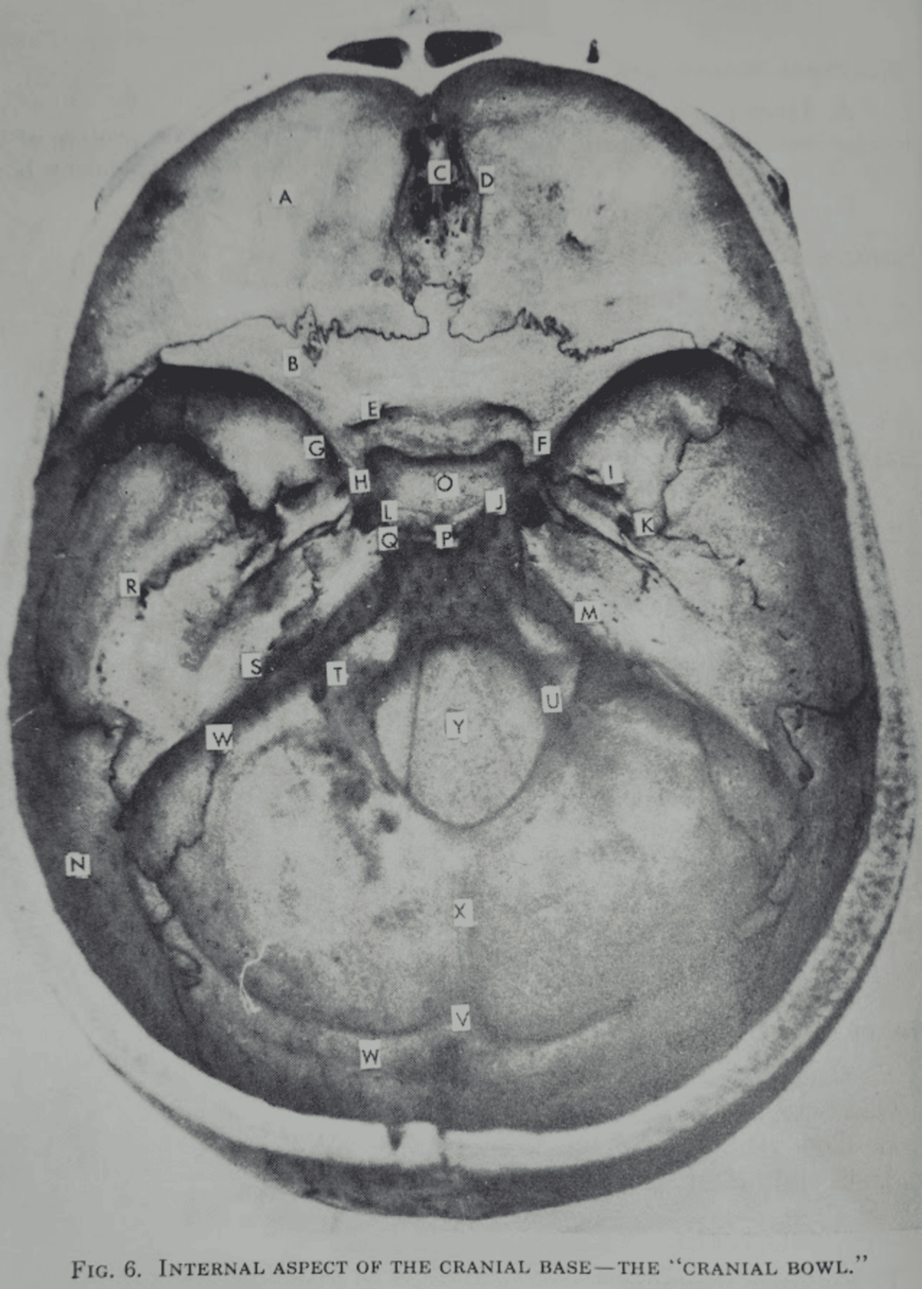
The internal aspect of the cranial base, often referred to as the "cranial bowl" in osteopathic cranial field theory. Source: Osteopathy in the Cranial Field Internal aspect of the cranial base—the "Cranial Bowl"." pg. 10 (https://archive.org/details/osteopathyincran0000will)

Dr. Sutherland's work in cranial osteopathy inspired a generation of practitioners who continued to develop and refine his techniques. Among his most notable students was Dr. Harold Magoun (1927-2011). Harold Magoun made significant contributions to the field through his writings and teachings. His book, *Osteopathy in the Cranial Field*, remains a seminal text in the study of cranial anatomy (Figure 6) and osteopathic medicine. Magoun's work helped to codify Sutherland's techniques and ensured that they would be passed on to future generations of osteopathic practitioners.

**Figure 7:**
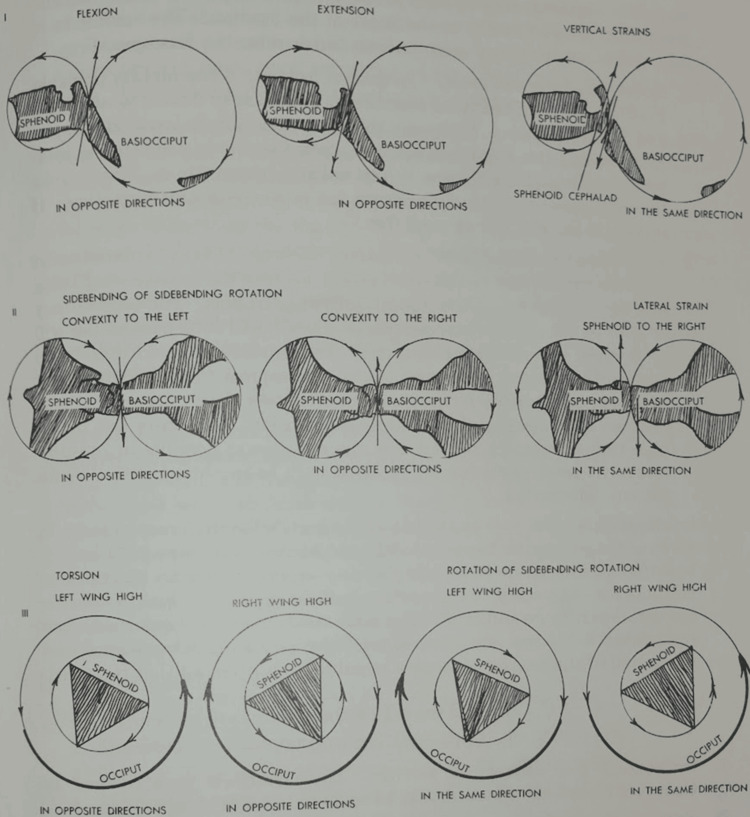
Magoun's crafted images designed to represent the movement patterns that are fundamental to diagnosing and treating cranial dysfunctions. Source: Osteopathy in the Cranial Field Fig. 54. Diagnostic summary of sphenobasilar lesions. pg. 141 (https://archive.org/details/osteopathyincran0000will)

Magoun would declare that cranial lesions are named (Figure 7) according to the direction of movement that is less restricted and classified lesions according to their severity from zero to four plus - from no to excessive mobility. He goes on to distinguish treatment principles for potential pathologies in the cranial region, including issues with nerves, membranes, CSF, and blood circulation. He explains the goal of treatment is to achieve balanced membranous tension, known as the point of balanced membranous tension, by guiding the flow of CSF [11]. Sutherland's influence is found throughout many chapters.

Sutherland’s liquid light vision: the "breath of life" and cranial motion

Although the exact source is unclear, it is widely reported that Dr. Sutherland often said,

Within that cerebrospinal fluid, there is an invisible element that I refer to as the ‘Breath of Life.’ I want you to visualize this Breath of Life as a fluid within this fluid, something that does not mix, something that has potency as the thing that makes it move. Is it necessary to know what makes the fluid move? Visualize a potency, an intelligent potency, that is more intelligent than your own human mentality.

While the exact origins of Sutherland’s quotes and references to "the hole in the tree" may be ambiguous, their symbolic value in conveying his revolutionary ideas is undeniable. These anecdotes, whether rooted in personal reflection or passed down through oral tradition, encapsulate his holistic approach to understanding the body. His work, particularly his exploration of cranial mobility, laid the groundwork for a new era in osteopathic medicine, challenging conventional medical wisdom and offering a fresh perspective on how the body's interconnected systems function.

His research revealed that cranial bones, once thought to be rigid and immovable in adults, exhibit subtle movement. This discovery fundamentally altered the understanding of cranial anatomy and its relationship to the body’s overall health. These insights were far from theoretical musings - Sutherland’s clinical applications demonstrated the profound therapeutic potential of addressing cranial mobility. His work, in fact, showed that disorders seemingly unrelated to the skull, including certain neurological, respiratory, and even gastrointestinal conditions, could be alleviated or mitigated through cranial manipulation. By focusing on the body’s natural ability to heal and self-regulate, Sutherland integrated anatomy, physiology, and the inherent capacity for balance into a coherent and effective therapeutic model.

 Modern impact: a legacy that endures

Today, William Sutherland's approach to OCMM is taught to physicians, dentists, and other healthcare professionals around the world. The Osteopathic Cranial Academy, founded in 1946, continues to promote Sutherland's teachings and offers advanced training in cranial osteopathy [12]. The Sutherland Cranial Teaching Foundation, established in his honor, provides a 40-hour course that delves into the intricacies of cranial manipulation, ensuring that his methods are passed on to new generations of practitioners [13]. Remarkably, Magoun served as Executive Vice President from its inception until 1972.

In addition to these institutions, Sutherland's influence is also evident in specialized centers such as the Osteopathic Center for Children and Families, which applies cranial osteopathy techniques to pediatric care [14]. Sutherland's pioneering work has not only endured but has also evolved, as modern practitioners continue to explore the applications of cranial osteopathy in various medical fields. His legacy is a testament to the lasting impact of his innovative ideas and his commitment to understanding the body's natural healing mechanisms.

In recent years, the relevance of Sutherland’s OCMM techniques has been bolstered by studies investigating its applications in modern healthcare. For instance, ongoing research in San Diego explores the use of osteopathic manipulative treatment (OMT) for infants with plagiocephaly, highlighting cranial techniques’ role in addressing skull asymmetry and enhancing patient outcomes [15]. Similarly, studies on pediatric upper respiratory infections (URI) have examined how cranial OMT may support respiratory function and reduce symptom severity in children [16]. These studies illustrate how Sutherland's concepts are being re-evaluated and adapted to contemporary medical challenges, blending traditional cranial principles with evidence-based practices. By bridging historical teachings with modern research, practitioners continue to validate and expand Sutherland’s impact on patient care.

## Conclusions

The practical implications of Dr. Sutherland's work have resonated through generations, encouraging countless practitioners to further investigate and refine cranial techniques. His ability to bridge anatomical knowledge with the body’s innate healing mechanisms not only broadened the scope of osteopathic practice but also set a foundation for more holistic approaches to healthcare. This integration of science and the body's self-regulatory capabilities has left an indelible mark on medicine and will continue to inspire future generations of practitioners.

Through the ongoing exploration of cranial manipulation, Sutherland's influence endures. His pioneering efforts invite modern practitioners to push the boundaries of osteopathic medicine, uncovering new possibilities for patient care. His work has forever shaped the field, fostering an enduring legacy of innovation, compassion, and commitment to understanding the body’s true potential for healing. The countless practitioners continuing to explore and expand upon his concepts ensure that his legacy remains not only relevant but central to the evolution of cranial manipulation.
